# Strontium-loaded titania nanotube arrays repress osteoclast differentiation through multiple signalling pathways: *In vitro* and *in vivo* studies

**DOI:** 10.1038/s41598-017-02491-9

**Published:** 2017-05-24

**Authors:** Baoguo Mi, Wei Xiong, Na Xu, Hanfeng Guan, Zhong Fang, Hui Liao, Yong Zhang, Biao Gao, Xiang Xiao, Jijiang Fu, Feng Li

**Affiliations:** 1 Department of Orthopaedic Surgery, Tongji Hospital, Tongji Medical College, Huazhong University of Science and Technology, Wuhan, 430030 China; 2 The State Key Laboratory of Refractories and Metallurgy, School of Materials and Metallurgy, Wuhan University of Science and Technology, Wuhan, 430081 China; 3 Institute of Biology and Medicine, Wuhan University of Science and Technology, Wuhan, 430065 China

## Abstract

The loosening of implants is an important clinical issue, particularly for patients with osteoporosis. In these patients, an implant should preferably both promote osteoblast differentiation and repress osteoclastic resorption. In the present study, we fabricated coatings containing TiO_2_ nanotubes (NTs) incorporated with strontium (Sr) on titanium (Ti) surfaces through hydrothermal treatment. The amount of loaded Sr was controlled by hydrothermally treating the samples in a Sr(OH)_2_ solution for 1 and 3 h (samples NT-Sr1h and NT-Sr3h, respectively) and found that both types of NT-Sr samples inhibited osteoclast differentiation by reducing the expression of osteoclast marker genes. Additionally, this inhibitory effect was mainly attributed to suppression of RANKL-induced activation of nuclear factor-κB (NF-κB). Moreover, NT-Sr also inhibited the Akt and nuclear factor of activated T-cell cytoplasmic 1 (NFATc1) signalling pathways. Interestingly, we also found that NT-Sr promoted RANKL-induced extracellular signal-regulated kinase (ERK) phosphorylation. Using ovariectomised rats as a model, we observed that NT-Sr prevented bone loss *in vivo*. In conclusion, our findings demonstrate that NT-Sr might effectively inhibit osteoclast differentiation by repressing the NF-κB and Akt/NFATc1 pathways and by negatively regulating the ERK pathway *in vitro* and *in vivo*.

## Introduction

Osteoporosis is a metabolic disease characterised by a reduction in bone mass due to an imbalance between bone formation and resorption^1, 2^. Patients with severe osteoporosis are more likely to experience fractures, and the pathological features of this disease include osteopenia, degradation of the bone tissue microstructure, and increased bone fragility. With the increased ageing of the global population, osteoporosis has become a major cause of fixation failure due to poor implant osseointegration. After these patients undergo surgery, poor osseointegration of the implant increases the likelihood of postoperative complications, such as poor primary stability of the implant, initial loosening, and impaired fixation strength in different regions^3^.

Titanium (Ti) and its alloys are widely used for orthopaedic implants because of their high corrosion resistance, biocompatibility, and mechanical properties^4, 5^. However, implant failure associated with poor osseointegration is a serious complication and usually necessitates repeated surgeries and/or implant removal, which incur additional costs to the patient^6^. To solve this clinical dilemma, researchers have explored the surface modification of biomaterials^7^, including surface nanotopography^8, 9^. In particular, TiO_2_ nanotubes (NTs) fabricated on Ti implant surfaces by electrochemical anodisation are attracting increased attention due to their excellent biological properties^10^ and the fact that their dimensional scale is similar to that of bone collagen fibrils^10, 11^. TiO_2_-NTs with different NT diameters were recently shown to enhance bone cell activity *in vitro*, promote implant osseointegration, and inhibit osteoclastogenesis^12^. Moreover, a significant advantage of TiO_2_-NTs is that they can serve as an excellent delivery platform for drugs, including antibacterial agents, growth factors, and inorganic bioactive elements^13, 14^. Multiple trace elements (Sr, Zn, and Ag) have been loaded into TiO_2_-NTs, and the resulting NTs were shown to facilitate osseointegration and antibacterial action^15^. TiO_2_-NTs could also be used as a scaffold for enabling the slow release of therapeutic agents *in situ* ^16^.

Strontium (Sr) is a trace but necessary element in bone. Sr-containing drugs, such as strontium ranelate (SrRan), are widely used as anti-osteoporosis drugs in the clinic because Sr exerts a significant anti-osteoporosis effect and decreases the risk of bone fracture in osteoporotic patients^17–19^. Sr has been reported to increase bone formation by osteoblasts and reduces bone resorption by inhibiting osteoclast differentiation *in vitro* ^20^. A recent study experimentally incorporated Sr into biomaterials such as Ti, hydroxyapatite, and mesoporous bioactive glass to enhance osseointegration^21–23^. TiO_2_-NTs loaded with Sr (NT-Sr) on Ti implants have been shown to enhance long-term osteogenic differentiation, and NT-Sr structures with different tube diameters enhance osteogenic differentiation to different degrees in rat bone mesenchymal stem cells^24^.

Osteoclasts are multinucleated cells derived from monocyte-macrophage lineage precursor cells and play an important role in bone development, growth and remodelling^25^. The formation and function of osteoclasts are mainly regulated by receptor activator of NF-κB (RANK) ligand (RANKL) and macrophage colony-stimulating factor (M-CSF)^26^. M-CSF promotes the survival and proliferation of osteoclast precursors and stimulates RANK expression^27, 28^. RANKL binds to its receptor RANK on osteoclast precursors and mature osteoclasts, leading to the recruitment of tumour necrosis factor (TNF) receptor-associated factors (TRAFs) and then activating the NF-κB, MAPK (ERK, JNK, and p38), Akt and nuclear factor of activated T-cells cytoplasmic 1 (NFATc1) signalling pathways^29–31^.

Many research results have confirmed that NT structures loaded with Sr on their surfaces can promote osteoblast differentiation and induce mineralisation. However, to the best of our knowledge, their effects on osteoclast differentiation and the related molecular mechanisms have not yet been reported. In this study, we constructed a TiO_2_-NT coating loaded with different concentrations of Sr and analysed the effects of the resulting NT-Sr samples on cell adhesion, proliferation, and differentiation and the function of osteoclasts. We also investigated the role of NT-Sr implants in an ovariectomised rat model and intensively examined the multiple signalling pathways involved in this process.

## Results

### Sample characterisation and analysis

**Figure 1:**
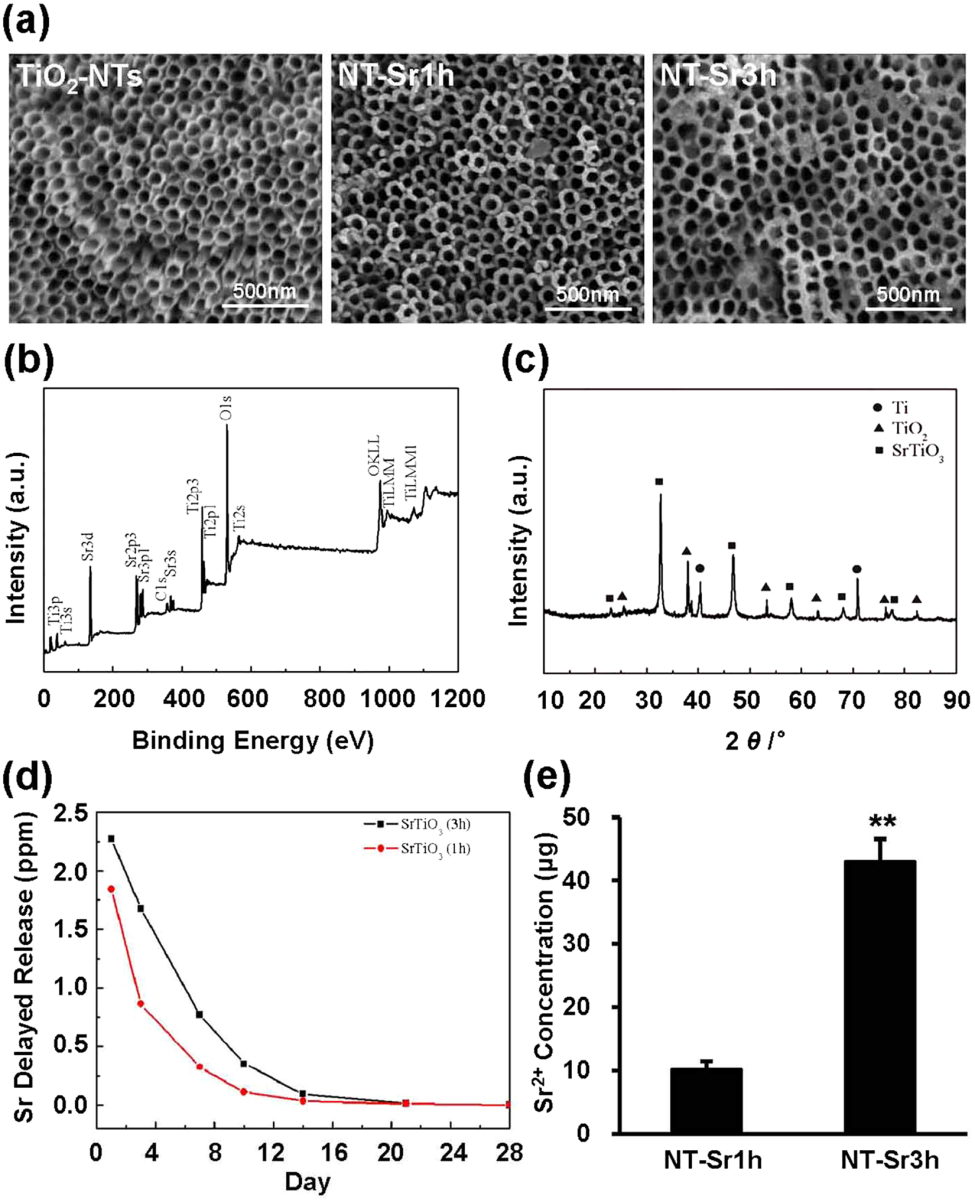
Microstructure and surface chemistry characterisation of the samples. (**a**) FE-SEM images of TiO_2_-NT, NT-Sr1h, and NT-Sr3h. The scale bar represents 500 nm. (**b**) XRD pattern of NT-Sr1h. (**c**) XPS survey spectra of NT-Sr1h. (**d**) Release of Sr from NT-Sr1h and NT-Sr3h into PBS at different time points. (**e**) Total Sr content of NT-Sr1h and NT-Sr3h, **p < 0.01. At least three independent experiments were analysed, and the data are presented as the means ± SDs.

The TiO_2_-NT samples produced by anodisation at 60 V were subjected to hydrothermal treatment for 1 and 3 h to form different Sr-containing TiO_2_-NT samples (denoted NT-Sr1h and NT-Sr3h, respectively). As shown in Fig. 1a, field-emission scanning electron microscopy (FE-SEM) images indicated that the average diameter of the TiO_2_-NT samples was approximately 100 nm, the width of the individual NTs was approximately 10 nm, and approximately 9 × 10^9^ TiO_2_ NT arrays were present on the Ti surfaces of each sample. The NT wall was rough and contained flakes in the TiO_2_-NT samples. However, the NT architecture was preserved in the NT-Sr samples. The flakes on top of the NT wall of NT-Sr3h were smoother than those on the NT wall of NT-Sr1h. As illustrated in Fig. 1b, the X-ray diffraction (XRD) patterns of the NT-Sr specimen contained peaks for Ti, anatase TiO_2_, and SrTiO_3_. In addition, Ti, O, and Sr peaks appear in the X-ray photoelectron spectroscopy (XPS) spectra of the NT-Sr specimen, as shown in Fig. 1c. These results show that TiO_2_-NT can load Sr ions and that the diameter between the samples is not significantly different.

### NT-Sr release Sr ions

The release of Sr was estimated by immersing the NT-Sr samples in 5 mL of PBS for up to four weeks. As shown in Fig. 1d, NT-Sr3h released a higher amount of Sr than NT-Sr1h. In general, NT-Sr showed an initial burst of Sr release, but 15 d later, the amounts of released Sr were relatively constant and slightly decreased. The total amount of released Sr from the NT-Sr1h and NT-Sr3h samples with the 1-cm^2^ coating were 10.2 and 43 μg, respectively (Fig. 1e).

### TiO_2_-NTs and NT-Sr promote protein adsorption

**Figure 2:**
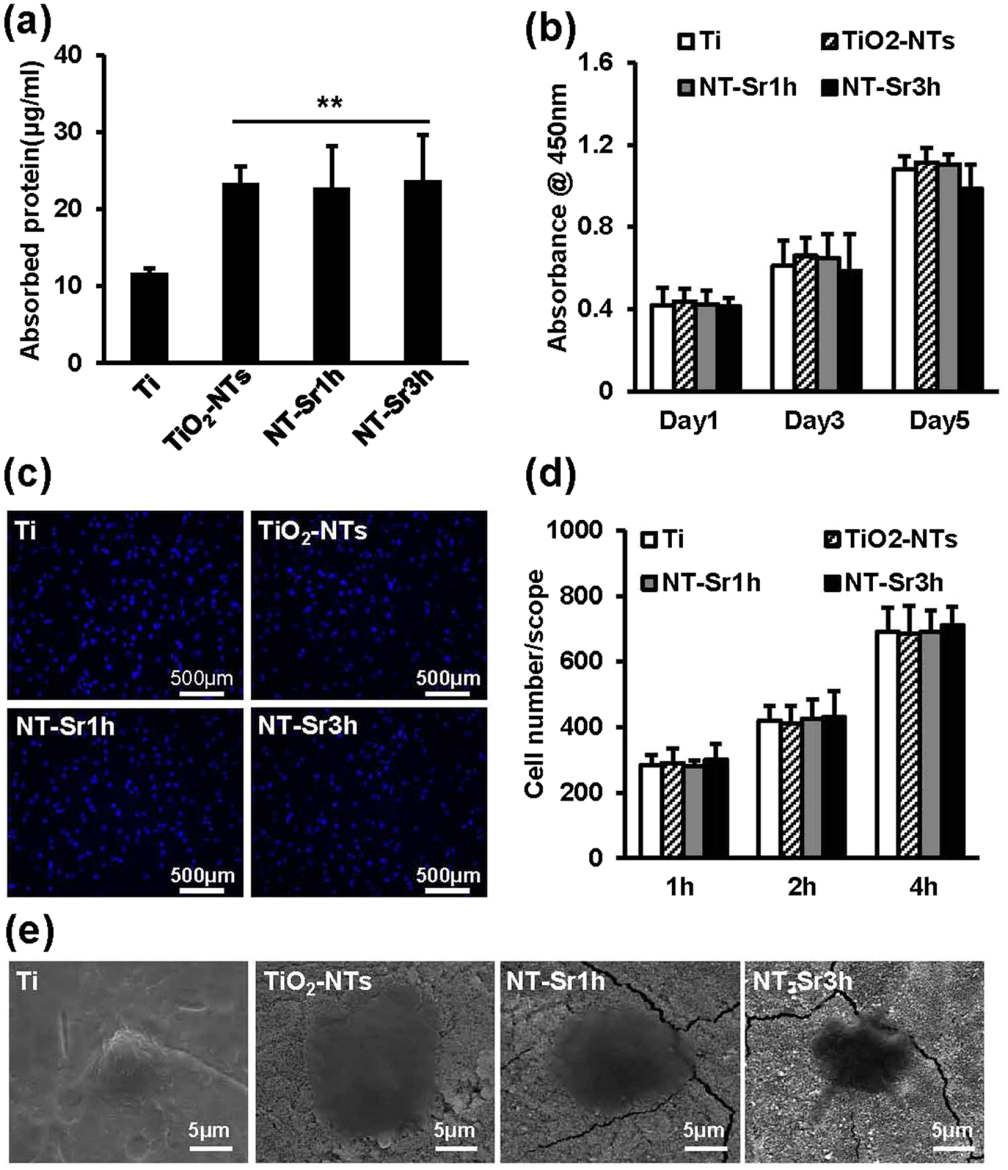
Biological properties of the samples. (**a**) Protein adsorption on different samples after immersion in α-MEM for 2 h. (**b**) RAW264.7 cell proliferation on the specimens after 1, 3, or 5 d of culture. Cell proliferation was measured by CCK-8. (**c**) After incubation for 2 h, RAW264.7 cells stained with DAPI. The scale bar represents 500 μm. (**d**) Initial adherent cell numbers at 1, 2, or 4 h. (**e**) After culturing for 2 d, the morphology of RAW264.7 cells on different samples was assessed by SEM images. The scale bar represents 5 μm. **p < 0.01 compared with the Ti group. At least three independent experiments were analysed, and the data are presented as the means ± SDs.

As revealed by the protein adsorption results shown in Fig. 2a, TiO_2_-NTs and NT-Sr adsorbed more protein than the Ti control. Slightly less protein was adsorbed onto the NT-Sr1h sample than onto the TiO_2_-NTs and NT-Sr3h samples, but no statistically significant differences were found among these groups. The results show that the amount of adsorbed protein depends mainly on the nanotopography.

### Effects of TiO_2_-NTs and NT-Sr on cell proliferation, adhesion and morphology

Cytotoxicity was assessed by CCK-8 on days 1, 3, and 5, and as shown in Fig. 2b, no appreciable differences in cell proliferation were detected among the samples. The initial numbers of adherent cells on the coated Ti samples were measured by DAPI staining, as shown in Fig. 2c, and the numbers of cells that adhered after culturing for 1, 2, and 4 h are shown in Fig. 2d. In general, no significant differences were observed between the samples cultured for 1, 2 and 4 h. Cell morphology was observed by SEM, and the data are shown in Fig. 2e. RAW264.7 cells spread poorly on the Ti surface, and the TiO_2_-NTs substantially facilitated cell extension. However, the NT-Sr-coated Ti surface resulted in poorer cell spreading than the TiO_2_-NT-coated Ti surface.

### NT-Sr inhibit osteoclast differentiation and activity

**Figure 3:**
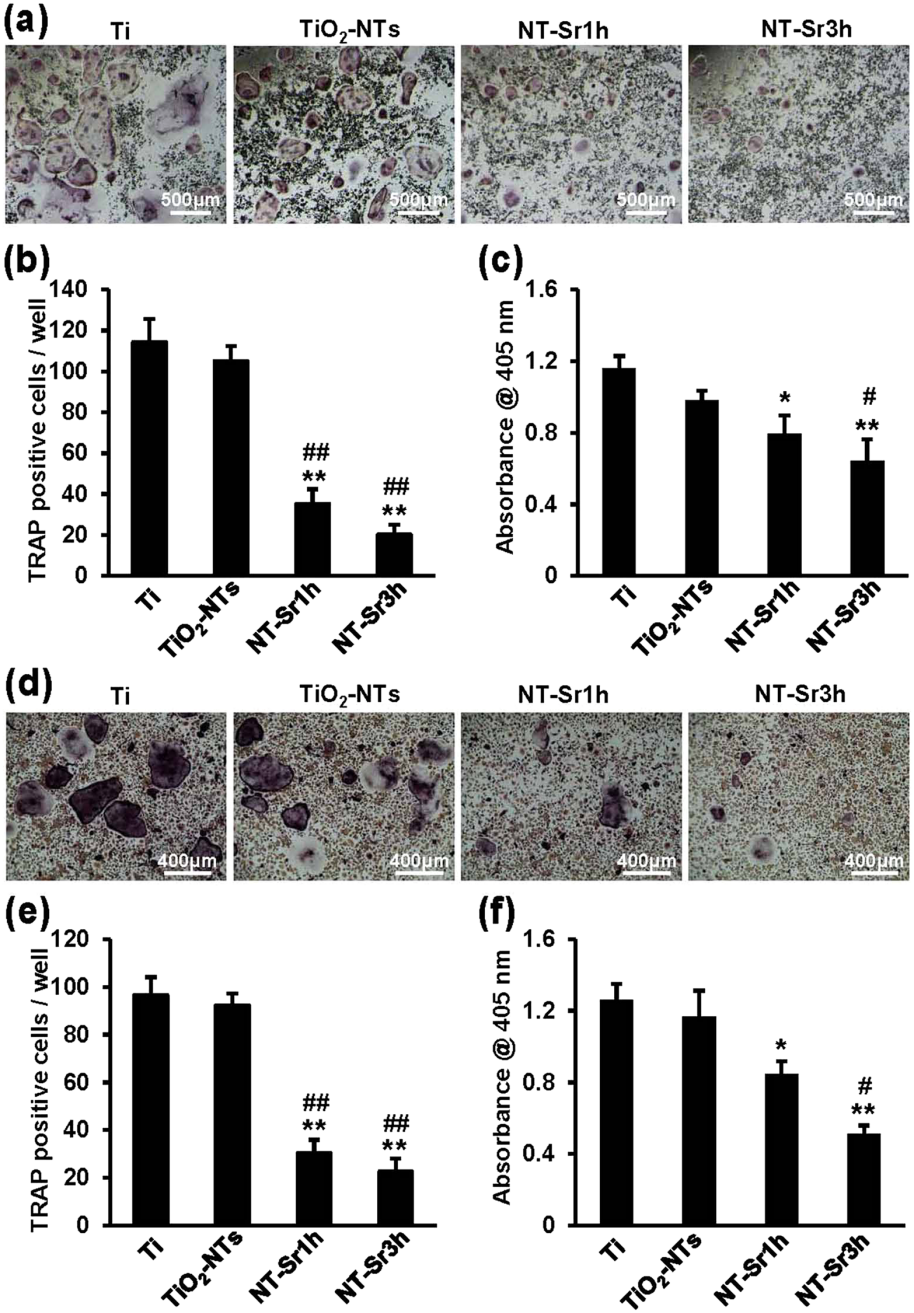
NT-Sr suppress osteoclast formation and activity. (**a**,**b**) RAW264.7 cells were induced with 50 ng/mL RANKL on different samples for 5 d and detected by TRAP staining. TRAP-positive cells with at least three nuclei were considered osteoclasts. The number of osteoclasts was quantified. The scale bar represents 500 μm (**a**). (**c**) RAW264.7 cells were induced with 50 ng/mL RANKL on different samples for 3 d, and TRAP enzyme activity was measured at OD 405 nm. (**d**,**e**) BMMCs were cultured in the presence of RANKL (50 ng/mL) and M-CSF (30 ng/ml) for 7 d. The cells were then fixed and stained for the TRAP assay. TRAP-positive cells with at least three nuclei were considered osteoclasts. The number of osteoclasts was quantified. The scale bar represents 400 μm (**d**). (**f**) BMMCs were induced with 50 ng/mL RANKL and M-CSF (30 ng/mL) on different samples for 3 d, and TRAP enzyme activity was measured at OD 405 nm. *^,^**p < 0.05 and 0.01, respectively, compared with the Ti group, and ^#,##^p < 0.05 and 0.01, respectively, compared with TiO_2_-NTs. At least three independent experiments were analysed, and the data are presented as the means ± SDs.

The effects of the different samples on osteoclast formation were evaluated by TRAP staining. As shown in Fig. 3a and d, the TRAP-positive multinuclear cells on the flat NT-Sr samples were smaller than those on the Ti and TiO_2_-NT samples, and this finding was obtained for both RAW264.7 cells and bone marrow mononuclear cells (BMMCs). As shown in Fig. 3b and e, the NT-Sr more significantly reduced the number of TRAP-positive multinuclear cells than the Ti and TiO_2_-NTs. Compared with Ti, TiO_2_-NTs also slightly repressed osteoclast formation, but this effect was not significant. We also detected TRAP activity using a TRAP enzyme assay kit and found that the activity of NT-Sr was significantly lower than that of the Ti samples (Fig. 3c and f). NT-Sr3h also more intensively decreased the TRAP activity of osteoclasts than TiO_2_-NTs; however, the differences between NT-Sr1h and TiO_2_-NTs and between TiO_2_-NTs and Ti were not statistically significant. These findings indicate that NT-Sr inhibit osteoclast formation and activity.

### NT-Sr decrease actin rings and resorption pits

**Figure 4:**
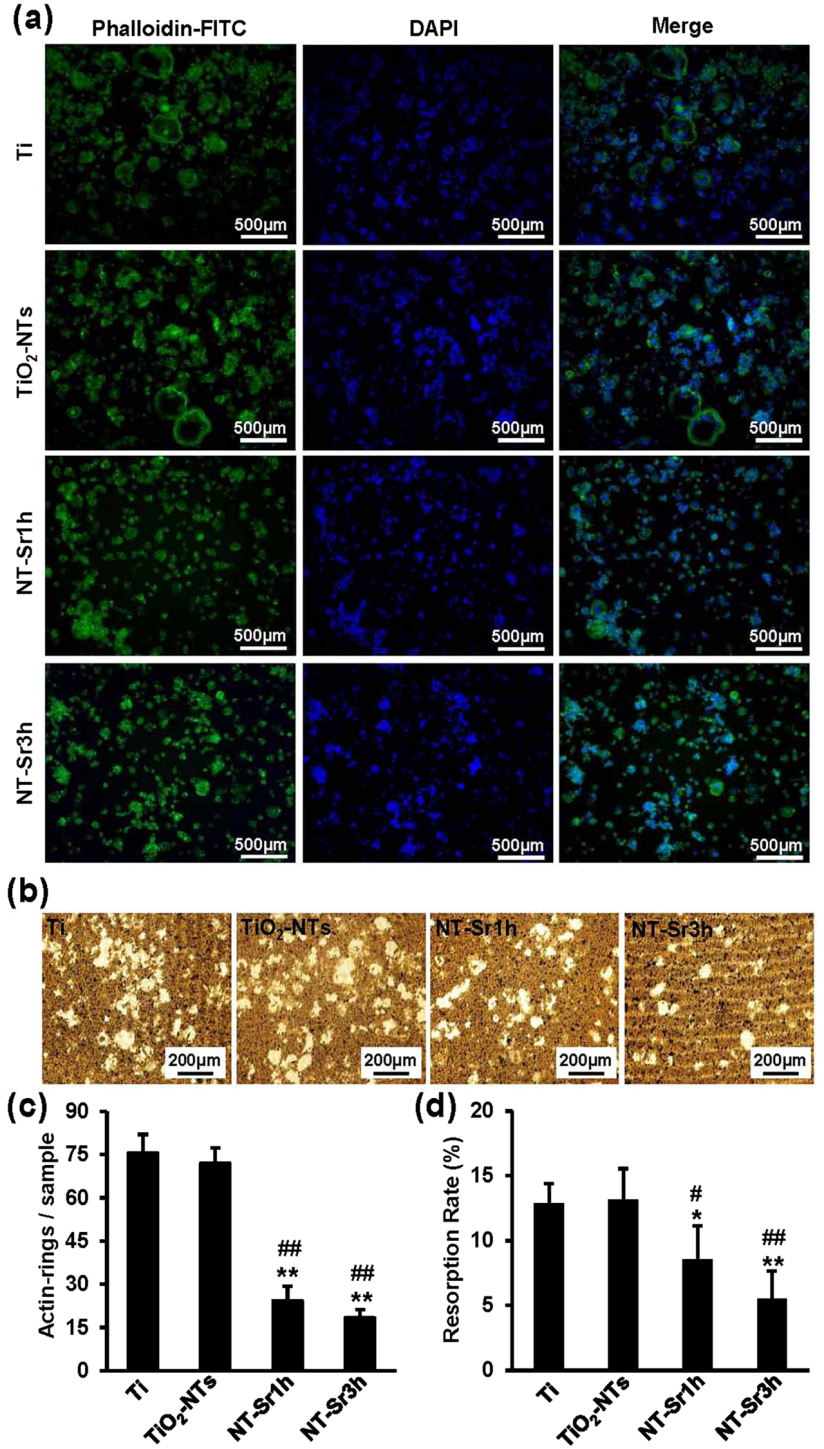
NT-Sr inhibit osteoclast formation and function. (**a**,**c**) RAW264.7 cells were incubated with 50 ng/mL RANKL on different samples. After culturing for 5 d, the cells were fixed, F-actin ring staining was performed, and the cells were examined by fluorescence microscopy. The number of F-actin rings was quantified. The scale bar represents 500 μm (**a**). (**b**) RAW264.7 cells were cultured on the Corning Osteo Assay Surface and incubated with 50 ng/mL RANKL for 5 d. The medium was then changed to conditioned medium, and the cells were cultured with 50 ng/mL RANKL for another 3 d. The cells were removed, and the resorbed areas were observed under a scanning electron microscope. The scale bar represents 200 μm. (**d**) The resorption areas are presented as percentages of the total well areas. *^,^**p < 0.05 and 0.01, respectively, compared with the Ti group, and ^#,##^p < 0.05 and 0.01, respectively, compared with TiO_2_-NTs. At least three independent experiments were analysed, and the data are presented as the means ± SDs.

We also assessed osteoclast formation on the specimens through immunofluorescence staining (Fig. 4a). Multinuclear cells containing an F-actin ring and at least three nuclei were surrounded by mononuclear cells. Osteoclasts with different sizes were detected on the different types of specimens. Giant cells were observed on the Ti sample, whereas the TiO_2_-NT specimens showed a markedly lower number of multinuclear cells. Cell induction on the NT-Sr specimens gradually decreased the number and size of the multinuclear cells. As shown in Fig. 4c, compared with the Ti and TiO_2_-NTs, the NT-Sr more significantly decreased the number of F-actin rings. Although NT-Sr markedly repressed osteoclast formation, whether these nanotubes affected mature osteoclast function was unknown. Therefore, we determined whether this suppression of osteoclast formation influenced the bone-resorbing function of osteoclasts. As shown in Fig. 4b, we found significantly fewer resorption pits in the NT-Sr group than in the Ti and TiO_2_-NT groups. The quantification of the resorption pits revealed that the cells on the NT-Sr samples formed approximately 10% fewer resorption areas than those on the Ti and TiO_2_-NT samples (Fig. 4d). These results indicate that NT-Sr inhibit the formation and function of osteoclasts.

### NT-Sr decrease the expression of osteoclast marker genes

**Figure 5:**
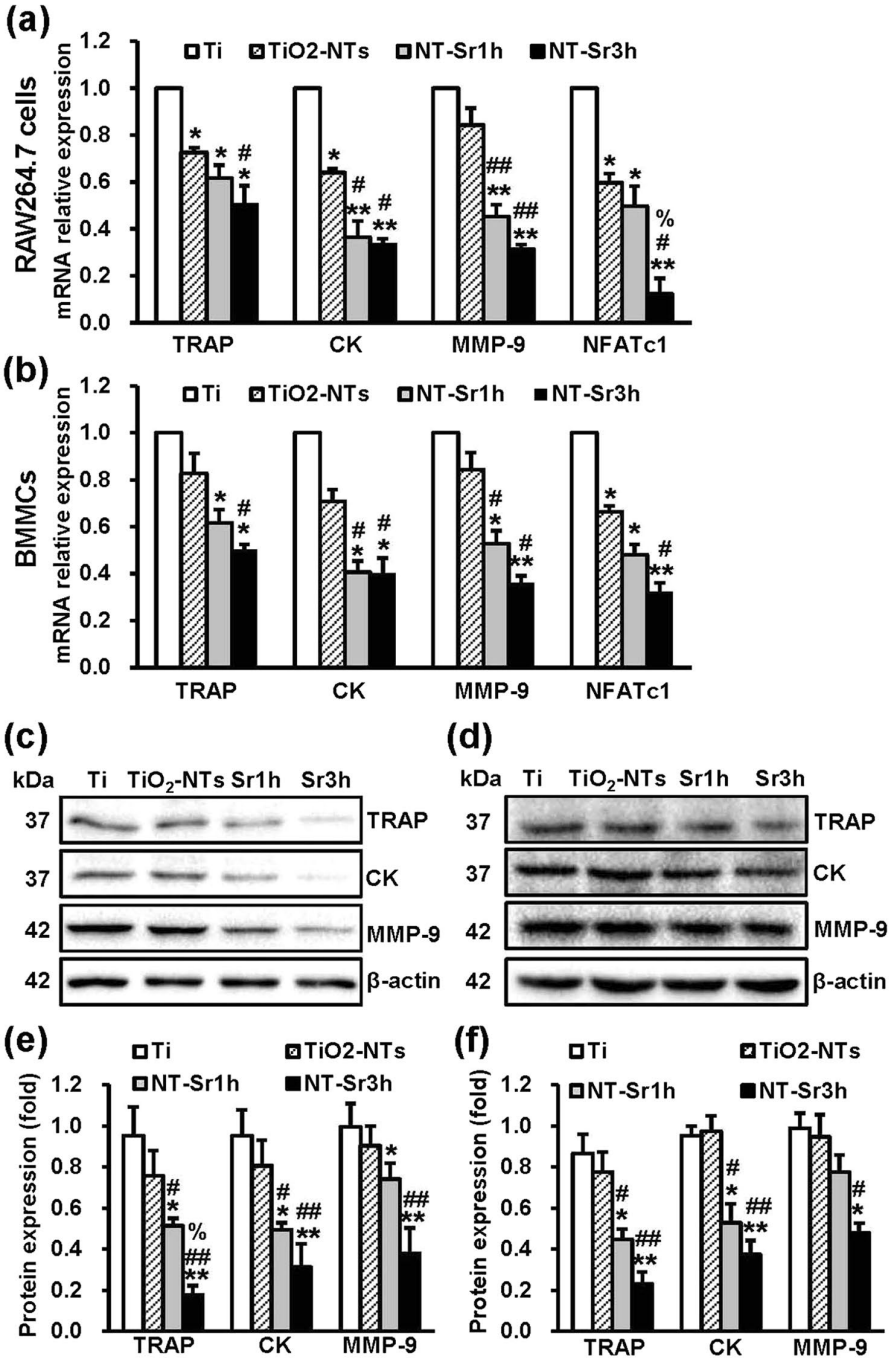
NT-Sr repress osteoclast-specific genes. RAW264.7 cells (**a**) and mouse BMMCs (**b**) were cultured on different samples and induced with 50 ng/mL RANKL and 30 ng/mL M-CSF (for BMMCs), and the total RNA was then collected. The relative mRNA expression levels of osteoclast-specific genes (TRAP, CK, MMP-9, and NFATc1) were assessed by qRT-PCR. For total protein extraction, RAW264.7 cells (**c**,**e**) and mouse BMMCs (**d**,**f**) were cultured on different samples in the presence of RANKL (50 ng/mL) and M-CSF (30 ng/ml, for BMMCs). The protein expression of osteoclast markers (TRAP, CK, and MMP-9) was then detected by immunoblotting. An antibody to β-actin was used as a loading control. A quantitative analysis of the band densities was performed, and the band densities were normalised to the loading control. Full-length blots are presented in Supplementary Figure 2. Sr1h and Sr3h represent NT-Sr1h and NT-Sr3h, respectively. *^,^**p < 0.05 and 0.01, respectively, compared with the Ti group, ^#,##^p < 0.05 and 0.01, respectively, compared with TiO_2_-NTs, and ^%^p < 0.05 compared with the NT-Sr1h group. At least three independent experiments were analysed, and the data are presented as the means ± SDs.

To further detect the influence of the samples on osteoclast differentiation, we detected the mRNA expression of osteoclast marker genes, such as TRAP, cathepsin K (CK), NFATc1, and MMP-9, in mouse BMMCs and RAW264.7 cells. In general, the TiO_2_-NT and NT-Sr inhibited the expression of all marker genes, and the significant differences among the groups are shown in Fig. 5a and b. We also measured the protein expression levels of TRAP, CK, and MMP-9 to demonstrate the down-regulation of these osteoclast-specific proteins by the NT-Sr (Fig. 5c and d), and the significant differences among the groups are shown in Fig. 5e and f. These results further indicate that NT-Sr inhibit osteoclast differentiation.

### NT-Sr inhibit osteoclast differentiation through multiple pathways

**Figure 6:**
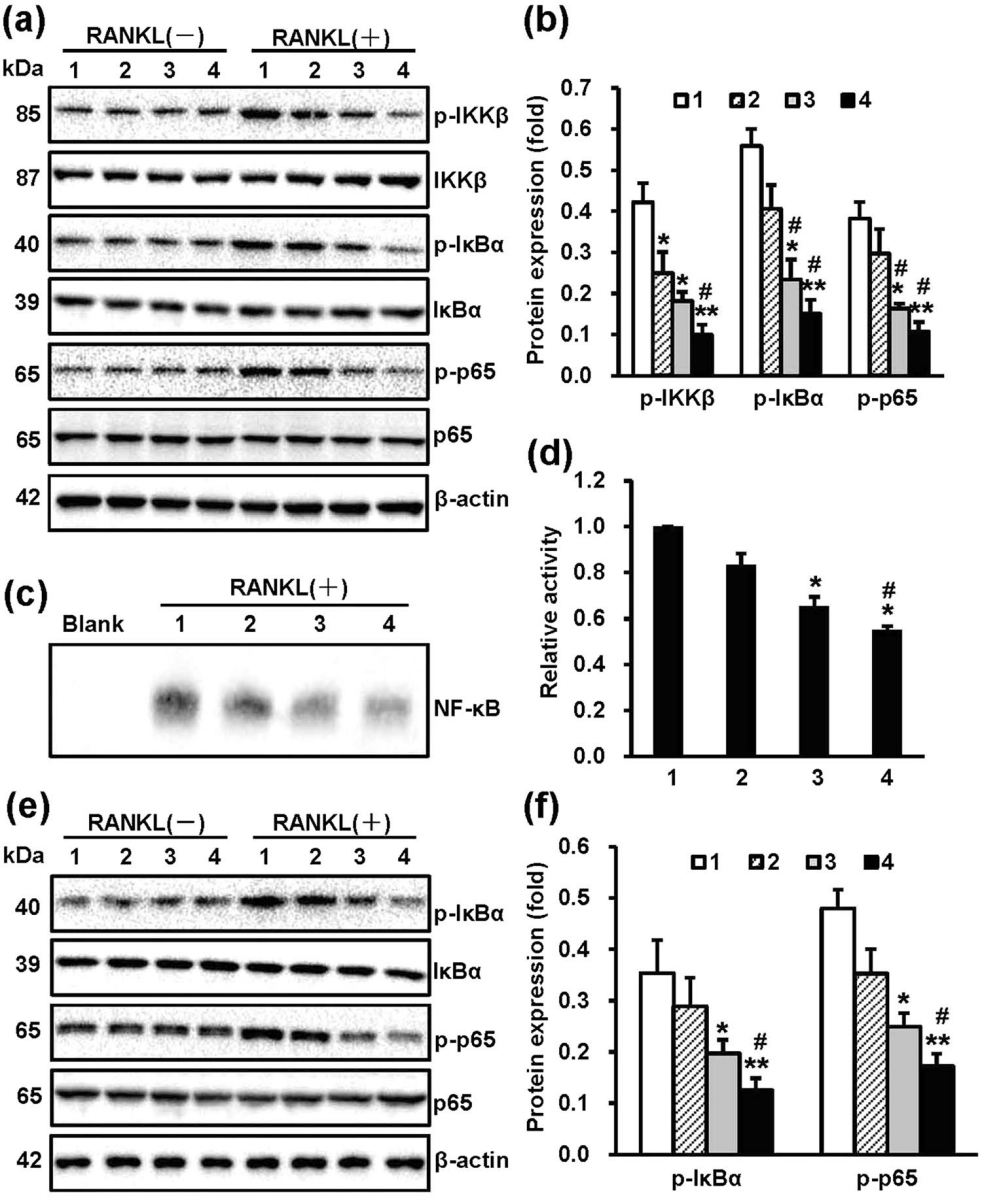
NT-Sr inhibit RANKL-induced NF-κB activation. After RAW264.7 cells (**a**,**b**) and mouse BMMCs (**e**,**f**) were cultured on different samples for 3 d, the cells were stimulated with or without 100 ng/mL RANKL for 30 min, and the total proteins were extracted for western blot analysis. The expression of proteins in the NF-κB pathway and the levels of p-IKKβ, p-IκBα, and p-NF-κBp65 were detected. Antibodies against β-actin, total IKKβ, IκBα, and NF-κBp65 were used as loading controls. A quantitative analysis of the band densities was performed, and the band densities were normalised to the loading controls. RAW264.7 cells (**c**,**d**) were cultured on different samples for 3 d and stimulated with 100 ng/mL RANKL for 30 min. The nuclear extracts were then collected, and the DNA-binding activity of NF-κB was detected by electrophoretic mobility shift assay (EMSA). A quantitative analysis of the band densities was performed, and the band densities were normalised to the loading controls. Full-length blots are presented in Supplementary Figure 3. p-p65 and p65 represent p-NF-κBp65 and NF-κBp65, respectively; and the numbers 1, 2, 3 and 4 in the figure represent Ti, TiO_2_-NTs, NT-Sr1h and NT-Sr3h, respectively. *^,^**p < 0.05 and 0.01, respectively, compared with the Ti group, and ^#^p < 0.05 compared with TiO_2_-NTs. At least three independent experiments were analysed, and the data are presented as the means ± SDs.

**Figure 7:**
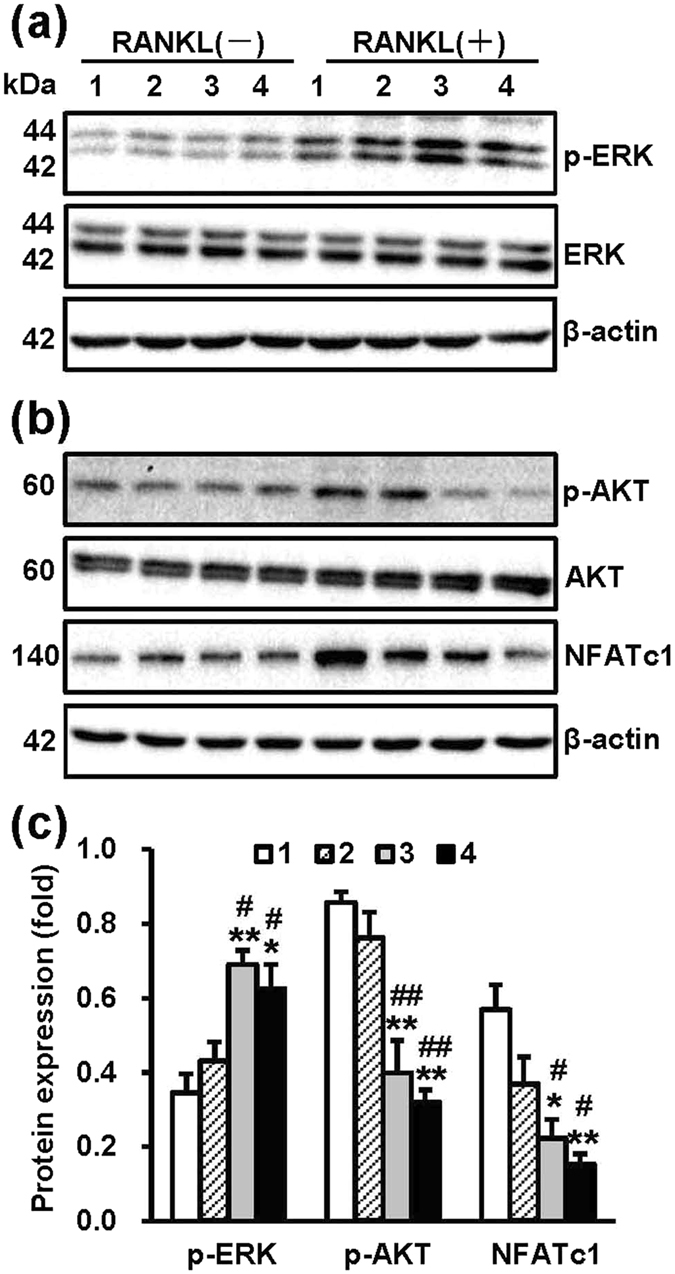
NT-Sr promote RANKL-induced phosphorylation of ERK and inhibit the Akt/NFATc1 pathway. RAW264.7 cells were cultured on different samples for 3 d and stimulated with or without 100 ng/mL RANKL for 30 min, and the total protein was then collected for immunoblot analysis. (**a**) RANKL-induced phosphorylation of ERK, (**b**) p-Akt and NFATc1 were detected using β-actin, total ERK and Akt as loading controls. (**c**) A quantitative analysis of the band densities was performed, and the band densities were normalised to the loading controls. Full-length blots are presented in Supplementary Figure 4. The numbers 1, 2, 3 and 4 in the figure represent Ti, TiO_2_-NTs, NT-Sr1h and NT-Sr3h, respectively. *^,^**p < 0.05 and 0.01, respectively, compared with the Ti group, and ^#,##^p < 0.05 and 0.01, respectively, compared with TiO_2_-NTs. At least three independent experiments were analysed, and the data are presented as the means ± SDs.

To explore whether NT-Sr suppress osteoclast differentiation via repression of the NF-κB pathway, we assessed the activation of NF-κB in RAW264.7 cells through western blot and EMSA assays. As shown in Fig. 6a and b, the NT-Sr suppressed RANKL-induced phosphorylation of IKKβ, IκBα, and NF-κBp65. The NT-Sr significantly impaired NF-κB DNA-binding activity, and the significant differences among the groups are shown in Fig. 6c and d. We then collected the total proteins from BMMCs and assessed the activation of NF-κB. The NT-Sr down-regulated RANKL-induced phosphorylation of IκBα and NF-κBp65 (Fig. 6e and f). Interestingly, the results shown in Fig. 7a indicate that the NT-Sr promoted ERK activation by RANKL. We then examined the phosphorylation of Akt and NFATc1 in RAW264.7 cells by western blot assays, and as shown in Fig. 7b, NT-Sr inhibited RANKL-induced phosphorylation of Akt and NFATc1 protein expression. The results suggest that NT-Sr might prevent the RANKL-induced activation of the Akt/NFATc1 pathway while inhibiting osteoclast differentiation. The significant differences among the groups are shown in Fig. 7c.

### NT-Sr prevent bone loss in OVX rats

**Figure 8:**
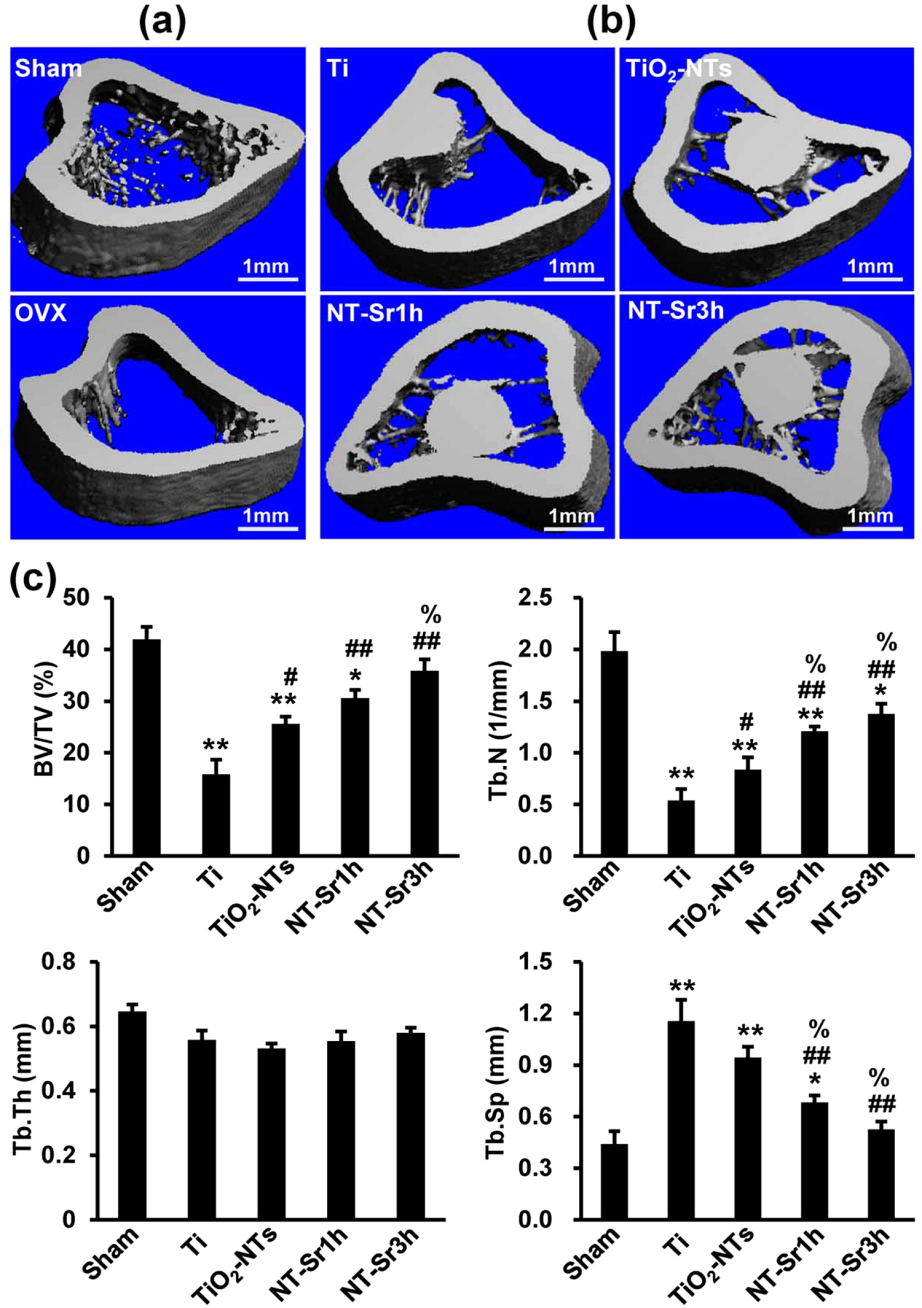
NT-Sr inhibit OVX-induced partial loss of bone mass. (**a**) Micro-computed tomography (µCT) images of the proximal tibia from sham and OVX groups. (**b**) µCT images of the proximal tibia from the different implants groups. The scale bar represents 1 mm (**a**,**b**). (**c**) BV/TV, Tb.N, Tb.Th, and Tb.Sp were analysed. *^,^**p < 0.05 and 0.01, respectively, compared with the sham group, ^#,##^p < 0.05 and 0.01, respectively, compared with the Ti group, and ^%^p < 0.05 compared with TiO_2_-NTs. The data are presented as the means ± SDs with n = 12 per group.

**Figure 9:**
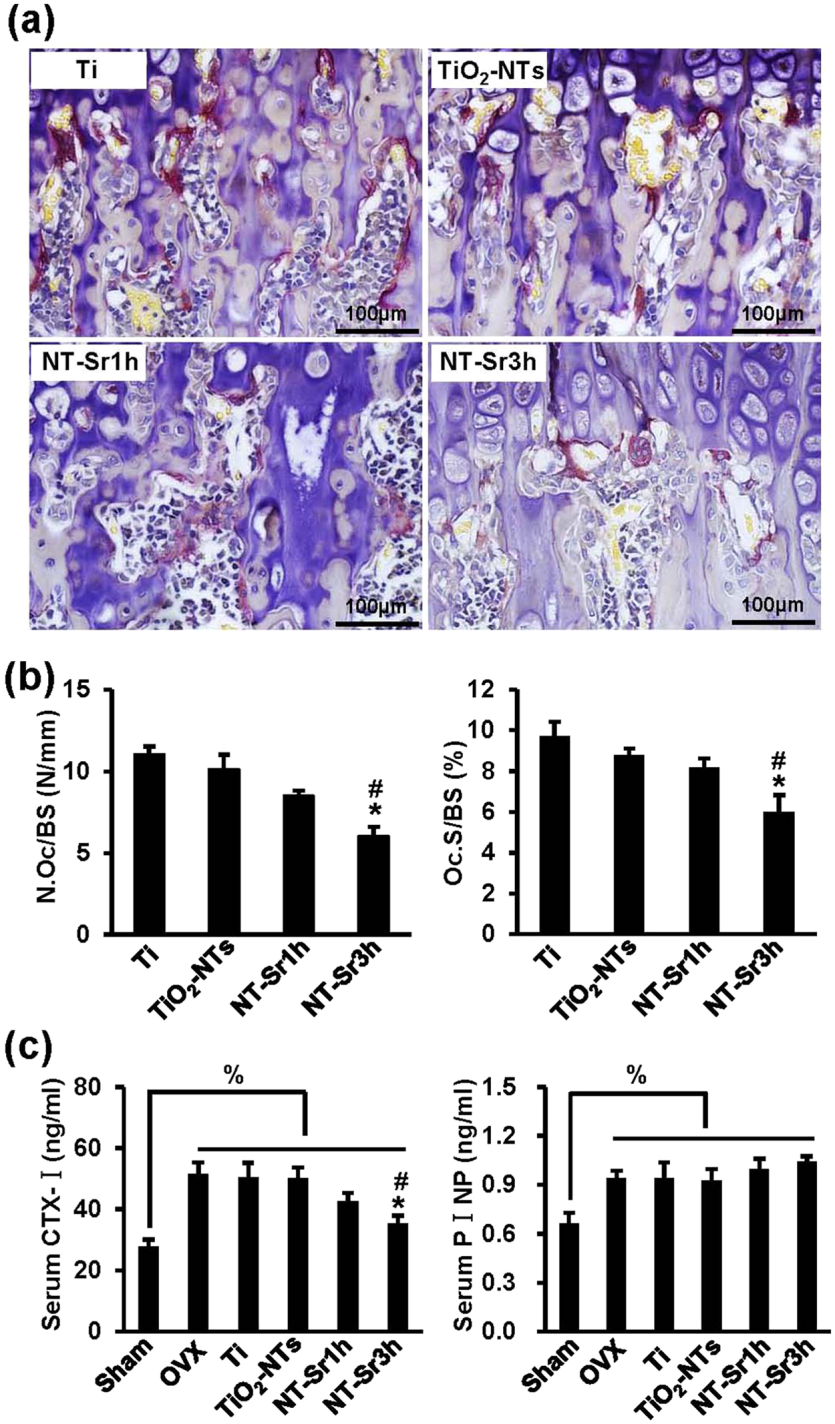
NT-Sr decrease osteoclast formation and function *in vivo*. (**a**,**b**) OVX rats were sacrificed eight weeks after implantation, sections of the metaphyseal regions of the proximal tibias (the area above the proximal end of the implant) were selected for TRAP staining, and osteoclastic parameters such as N.Oc/BS and Oc.S/BS were measured. The red multinucleated cells were considered osteoclasts. The scale bar represents 100 μm (**a**). Low-magnification images are presented in Supplementary Figure 5. (**c**) The serum levels of CTX-I and PINP were examined by ELISA. *p < 0.05 compared with the Ti group, ^#^p < 0.05 compared with TiO_2_-NTs, and ^%^p < 0.05. The data are presented as the means ± SDs with n = 12 per group.

To expound the effects of the samples on OVX-induced bone loss *in vivo*, we established osteoporosis models by removing the bilateral ovaries of rats. Eight weeks after the operation, the degree of trabecular bone was significantly reduced in the OVX rats compared with the sham-operated rats (Fig. 8a). The results shown in Fig. 8b provide information regarding the effects of the different implants on bone loss in the OVX rats. In general, more trabeculae was found around the NT materials, particularly around the implants loaded with Sr. The results of the quantitative analysis are shown in Fig. 8c. To investigate whether NT-Sr inhibit bone loss by inhibiting osteoclastogenic activity *in vivo*, we examined TRAP staining on tibial slices. As shown in Fig. 9a, the rats treated with NT-Sr displayed fewer and smaller TRAP-positive multinucleated cells than the OVX rats treated with Ti and TiO_2_-NTs. Histomorphometric analysis confirmed that the osteoclast number/bone surface (N.Oc/BS, N/mm) and osteoclast surface/bone surface (Oc.S/BS, %) were significantly decreased in OVX rats treated with NT-Sr3h compared with those treated with Ti and TiO_2_-NTs (Fig. 9b). We also detected the serum levels of procollagen I N-terminal peptide (PINP, a biochemical marker of bone formation) and type I collagen cross-linked C-terminal telopeptide (CTX-I), which is a serological marker of bone resorption (Fig. 9c) and found that the serum levels of CTX-I and PINP were generally increased in OVX rats compared with the sham control rats, whereas NT-Sr3h significantly decreased the CTX-I levels induced by OVX. In addition, NT-Sr increased the serum levels of PINP, but no statistically significant differences were detected among the groups. These findings suggest that NT-Sr effectively prevent bone loss by inhibiting osteoclastogenic activity *in vivo*.

## Discussion

The aseptic loosening of an implant from the surrounding bone tissue is a leading cause of orthopaedic implant failure^32^. A credible hypothesis for the occurrence of aseptic loosening is that poor osseointegration during the early period results in micromotion of the bone–implant interface^33^. This complication is much more common in osteoporotic patients. To overcome this challenge, surface modification strategies for orthopaedic implants have been studied intensively to improve the proliferation and differentiation of osteoblasts or inhibit osteoclast differentiation^34, 35^. A previous study demonstrated that NT-Sr could promote osteogenesis^24^. However, the ability of NT-Sr to repress osteoclast differentiation remains unexplored.

In the current study, we fabricated NT-Sr coatings in Sr solutions through a hydrothermal process. Sr overdose might lead to cytotoxicity^36^, and the results obtained in the present study, which are shown in Fig. 2b, indicate that the amount of Sr released from NT-Sr1h and NT-Sr3h did not affect the number of RAW264.7 cells after 1, 3 and 5 d of culture. However, the maximum concentration of Sr released from NT-Sr5h leads to considerable cytotoxicity (Supplementary Figure 1). Therefore, we observed the effects of the NT-Sr1h and NT-Sr3h samples on osteoclast differentiation. The proteins adsorbed on material surfaces play an important role in conveying the biological effects of the topographical cues^24^. Our study demonstrates that the TiO_2_-NTs and NT-Sr adsorb the same amount of proteins but more proteins than Ti. The results indicate that the amount of adsorbed protein depends mainly on the nanotopography. Consistent with a previous report^37^, our findings show that the number of initially adherent cells did not present an obvious difference among the different samples. In this study, we also found that RAW264.7 cells on the TiO_2_-NTs structure exhibited improved spreading, whereas NT-Sr markedly inhibited cell extension.

In the present study, we also examined the effects of NT-Sr on osteoclast differentiation *in vitro* and *in vivo* and found that NT-Sr, particularly NT-Sr3h, repress osteoclast formation, activity, and function *in vitro*. In addition, the µCT evaluation revealed more trabecular bone with the anodised implants, particularly the NT-Sr3h sample, than with Ti. Consistent with a previous study^38^, ovariectomy produced a significant increase in bone remodelling, in terms of both resorption (CTX-I) and formation (PINP). The current study also demonstrates that NT-Sr decreases the CTX-I levels induced by OVX. According to the above-mentioned results, we hypothesise that the local delivery of Sr could effectively prevent OVX-induced bone loss *in vivo*, and this effect becomes more obvious with a higher amount of released Sr.

The potential molecular mechanisms were discussed and analysed based on the NF-κB and Akt/NFATc1 signalling pathways as well as the ERK pathway. The NF-κB pathway, which plays a pivotal role in osteoclast differentiation, is well understood based on the findings from genetic and pharmacological studies^39^. The classical NF-κB signalling pathway involves activation of the IκB kinase (IKK) complex, which phosphorylates IκBα and targets it for ubiquitin-dependent degradation^40, 41^. Recent study has revealed that Sr suppresses Ti particle-induced osteoclast activation through of the NF-κB pathway^42^. Our results show that NT-Sr inhibits RANKL-induced activation of NF-κB by suppressing IκBα phosphorylation and prevents NF-κBp65 nuclear translocation. The results indicate that inhibition of the NF-κB-dependent pathway is one of the mechanisms underlying the anti-osteoclastogenic effect of NT-Sr.

The data from a series of previous *in vitro* experiments suggest that mitogen-activated protein kinases (MAPKs, including p38 MAPK, ERK1/2, and JNK) also play an important role in osteoclastogenesis^43, 44^. It has been reported that p38 MAPK plays a critical role in RANKL-induced osteoclast differentiation^45^. However, our study reveals that NT-Sr has almost no effect on the protein expression of p38 MAPK (Supplementary Figure 4). Interestingly, we found that NT-Sr promotes RANKL-induced phosphorylation of ERK in RAW264.7 cells. Similarly, the inhibition of ERK activity by an MEK inhibitor has been reported to not suppress but rather potentiate osteoclastogenesis^46^, suggesting that the ERK pathway negatively regulates osteoclastogenesis^47^. The results of an *in vitro* study indicated that JNK1 is at least partly involved in osteoclastogenesis^44^. Osteoclast progenitor cells derived from jnk1^−/−^ but not jnk2^−/−^ mice have been shown to have reduced potential for RANKL-stimulated osteoclastogenesis, suggesting that JNK1 but not JNK2 is important for efficient osteoclast differentiation^47^. In this study, we demonstrate that NT-Sr do not affect the level of RANKL-induced JNK phosphorylation (Supplementary Figure 4).

The Akt signalling pathway has been shown to regulate osteoclast survival and differentiation. The overexpression of Akt significantly enhances RANKL-induced osteoclast formation^48^. Akt phosphorylation is activated by the stimulation of both M-CSF and RANKL and plays a critical role in osteoclastogenesis by affecting the activation of both NF-κB and NFATc1^49^. PI3K/Akt activation leads to GSK3β phosphorylation, and this inhibition of GSK3β leads to the nuclear localisation of NFATc1, resulting in enhanced osteoclastogenesis^48^. In the present study, our observation that NT-Sr reduces Akt phosphorylation and NFATc1 protein expression indicates that low levels of Akt phosphorylation suppress NFATc1 expression, resulting in repressed osteoclast differentiation.

We systematically demonstrate that NT-Sr are able to inhibit osteoclast differentiation through the NF-κB and Akt/NFATc1 pathways and negative regulation of the ERK pathway *in vitro*. We further verified this hypothesis *in vivo* through a rat model of post-menopausal osteoporosis. The effects of NT-Sr on osteoclast differentiation *in vivo* and *in vitro* suggest that NT-Sr possess promising potential for future clinical translation.

## Methods

### Sample synthesis and characterisation

Ti foils (99.7% pure, Aldrich) were cut into 10 × 10 × 1-mm^3^ pieces, polished with SiC sandpaper (×200, ×600, ×800, ×1500, and ×2000), and ultrasonically washed sequentially with acetone, ethanol, and deionised water (DI water). TiO_2_ NT arrays were fabricated on Ti surfaces through electrochemical anodisation. The anodisation electrolyte ethylene glycol contained 0.5 wt% NH_4_F, 5 vol% H_2_O, and 5 vol% methyl alcohol (CH_3_OH), and anodisation was performed at 60 V for 30 min. The TiO_2_-NTs were then annealed at 450 °C, and 2 h later, the TiO_2_-NTs were placed in a 20 mM Sr(OH)_2_ solution and subjected to hydrothermal treatment at 200 °C for 1 or 3 h to generate SrTiO_3_ NT arrays. These specimens were ultrasonically washed with a 1 M HCl solution followed by DI water to remove the residual Sr(OH)_2_ and subsequently dried in air. The samples were characterised by FE-SEM (FEI Nova 450 Nano), XRD (Cu Kα radiation, Philips X’Pert Pro), and XPS (Thermo Fisher ESCALAB 250Xi) for evaluation of their surface morphology and chemical composition. We also fabricated Sr-containing cylindrical rod samples for internal experiments using the same anodisation, hydrothermal reaction, and annealing processes described above for the Ti foil. The diameter and length of the cylindrical rod samples were 0.8 mm and 1 cm, respectively.

### Determination of Sr release

The SrTiO_3_ samples were incubated at 37 °C for 1, 3, 7, 10, 14, 21, and 28 d with 5 mL of phosphate-buffered saline (PBS), and the PBS was changed every day. The delayed release of Sr in PBS was analysed through inductively coupled plasma atomic emission spectrometry (ICP-AES). The detection limit of the ICP analysis was 0.001 ppm. The standards used for Sr detection were gradient dilutions of Sr(OH)_2_ prepared in PBS. The emission line for Sr was 407.7 nm, and the total Sr contents were determined by ICP-AES.

### Protein adsorption assay

The protein adsorption assay was conducted in 1 mL of α-MEM containing 10% heat-inactivated foetal bovine serum (FBS, HyClone). The specimens were incubated at 37 °C for 2 h with the medium, and the specimens were then transferred to another new 24-well plate. After washing twice with PBS, the proteins adsorbed onto the samples were detached using 500 μL of 1% sodium dodecyl sulphate and measured with Micro BCA protein assay kit (Boster, Wuhan, China).

### Animals

The animal studies were approved by the Institutional Animal Research Committee of Tongji Medical College. The animal experimental procedures were performed in accordance with protocols approved by the Institutional Animal Care and Use Committee. C57/BL6 mice and Sprague Dawley rats were housed at 25 °C with 55% relative humidity and light/dark cycles. All animals were permitted free access to chow and tap water.

### Cell culture

We cultured BMMCs obtained from six- to eight-week-old C57BL/6 mice as described previously^50^. RAW264.7 cells were purchased from the Chinese Academy of Sciences. These two types of cells were maintained in α-MEM (HyClone) with 10% FBS, M-CSF (30 ng/mL for BMMCs), 100 μg/mL streptomycin and 100 U/mL penicillin.

### Cell adhesion

RAW264.7 cells were seeded on the samples, which were placed in a 24-well plate at 4 × 10^4^ cells/well. After incubation of the cells for 1, 2, or 4 h, the samples were washed with PBS. The attached cells on the samples were then fixed with 4% paraformaldehyde and stained with 4′,6-diamidino-2-phenylindole (DAPI). Images were captured from five random fields with a fluorescence microscope (Nikon, Japan), and the cell number in each field was counted.

### Cell proliferation

To assess cell proliferation, the cells were cultured on samples at a density of 2 × 10^4^ cells per well, and 1, 3, or 5 d after treatment, the degree of cell proliferation was measured using a Cell Counting Kit-8 assay (CCK-8, Beyotime, China).

### Cell morphology

To study the morphology of the cells on the samples, the cells were cultured on the samples at a density of 2 × 10^4^ cells per well. After the cells were cultured for 2 d, the samples were washed with PBS, and the cells on the samples were fixed with 3% glutaraldehyde. After 15 min, the cells on the samples were dehydrated with different concentrations of ethanol. After freeze-drying and coating with gold, the cell morphology was examined through SEM.

### Osteoclast formation and activity assays

RAW264.7 cells and mouse BMMCs were cultured with M-CSF (30 ng/mL for BMMCs) and RANKL (50 ng/mL) to induce osteoclast formation. For TRAP staining, the specimens were gently washed twice with PBS and transferred to a new 24-well plate. Mature osteoclasts were recovered from the specimens using 0.25% trypsin and seeded in new 24-well plates. After allowing the cells to adhere for 2 h, a TRAP staining kit (Sigma-Aldrich, Shanghai, China) was used to assess osteoclast formation. Cell images were taken with a microscope (Nikon ECLIPSE TE2000-S, Japan), and the cells with at least three nuclei were identified as osteoclasts. We also measured osteoclast activity using a TRAP enzyme assay kit (Sigma-Aldrich, Shanghai, China) according to the manufacturer’s instructions. Briefly, the specimens were gently washed twice with PBS, transferred to a new 24-well plate and lysed with 0.1% Triton X-100. The supernatant solution was collected, and TRAP enzyme activity was analysed at 405 nm using a colourimetric plate reader.

### Immunofluorescence staining and pit formation assays

To further evaluate osteoclast formation on the specimens, actin ring formation assays were performed as described previously^51^. The osteoclast contours and nuclei were visualised under a fluorescence microscope (Nikon), and images were captured from five random fields. To explore the influence of NT-Sr on osteoclast function, we performed pit formation assays as follows. To prepare the conditioned medium, the specimens were incubated with α-MEM containing 10% FBS. We cultured RAW264.7 cells on the Corning Osteo Assay Surface (Corning Incorporated Life Science, USA) and then treated the cells with RANKL for 5 d. After treatment, the medium was changed to conditioned medium containing RANKL, and after culturing for another 3 d, we washed the disc with 5% sodium hypochlorite and quantified the resorption area through image analysis (Bioquant Image Analysis, Nashville, TN, USA).

### Expression of osteoclast-specific mRNAs

The expression levels of osteoclast marker genes, such as TRAP, MMP-9, CK and NFATc1, were measured through quantitative real-time polymerase chain reaction (qRT-PCR). Briefly, the total RNA from RAW264.7 cells and BMMCs grown on the different samples was obtained using the TRIzol reagent (Invitrogen), and first-strand cDNA was synthesised from 2 μg of total RNA using a reverse transcriptase kit (Thermo Fisher Scientific). The templates were then amplified on CFX96 (Bio-Rad, CA, USA) using a Power SYBR Green PCR Master Mix (Thermo Fisher Scientific). The primers used for qRT-PCR are listed in Table 1.

**Table 1:** Oligonucleotides used for quantitative real-time PCR.

Target gene	Forward primer Reverse primer (5′ to 3′)	Reverse primer Reverse primer (5′ to 3′)
GAPDH	CTCCCACTCTTCCACCTTCG	TTGCTGTAGCCGTATTCATT
TRAP	GATGCCAGCGACAAGAGGTT	CATACCAGGGGATGTTGCGAA
CK	GAAGAAGACTCACCAGAAGCAG	TCCAGGTTATGGGCAGAGATT
MMP-9	CTGGACAGCCAGACACTAAAG	CTCGCGGCAAGTCTTCAGAG
NFATc1	CAACGCCCTGACCACCGATAG	GGGAAGTCAGAAGTGGGTGGA

### Western blot analysis

Immunoblotting was performed as described previously^52, 53^. Antibodies against TRAP (ab96372), MMP-9 (ab38898) and CK (ab19027) were purchased from Abcam and used at 1:500 dilution. The following primary antibodies were purchased from Cell Signalling Technology and used at 1:1000 dilution: phospho-ERK1/2 (p-ERK1/2), ERK1/2, phospho-Akt (p-Akt), Akt, phospho-IKKβ (p-IKKβ), IKKβ, phospho-IκBα (p-IκBα), IκBα, phospho-NF-κBp65 (p-NF-κBp65), NF-κBp65, and NFATc1. The antibody against β-actin (β-actin) and secondary antibodies were purchased from BOSTER (Wuhan, China, 1:5000 dilution).

### Electrophoretic mobility shift assay (EMSA)

The extraction of nuclear proteins and EMSA were performed as described previously^54^. A chemiluminescent EMSA kit (Pierce, USA) was used for detecting the DNA-binding activity of NF-κB. The consensus recognition site for NF-κB was 5′-AGTTGAGGGGACTTTCCCAGGC-3′. The membrane was then exposed using the ChemiDoc™ XRS + System with Image Lab™ Software (Bio-Rad, CA, USA).

### Animal model design

Ten-week-old female Sprague Dawley rats weighing approximately 250 g were randomly divided into six groups (Sham, OVX, OVX + Ti, OVX + TiO_2_-NTs, OVX + NT-Sr1h, and OVX + NT-Sr3h) with 12 rats in each group. Ovariectomy (OVX) was performed by removing the bilateral ovaries, and sham surgery was performed by identifying the bilateral ovaries. For the corresponding implant groups, a Ti, TiO_2_-NTs, NT-Sr1h, or NT-Sr3h implant was placed in the tibial medullary cavity, as described previously^55^. Briefly, after anaesthesia with pentobarbitone, an incision was made across the rat knee, and we then drilled a pilot hole in the intercondylar uplift and twisted a needle (0.8-mm diameter) from the proximal tibial metaphysis into the medullary canal to create a channel. We then inserted the implants into the medullary canal until they were under the growth plate. Eight weeks after surgery, all rats were anaesthetised and subjected to micro-computed tomography (μCT). The rats were then sacrificed for histological evaluation.

### Micro-computed tomography (μCT) analysis

After anaesthesia, the tibias with implants were subjected to μCT (vivaCT40, Scanco Medical, Switzerland). Image acquisition was performed at 70 kV and 114 μA with an integration time of 380 ms and a resolution of 20 μm. The volume of interest was designed as part of 50 slices starting from 5 mm under the growth plate. The bone volume/tissue volume (BV/TV), trabecular separation (Tb.Sp), trabecular thickness (Tb.Th), and trabecular number (Tb.N) were analysed using the built-in software of the μCT instrument.

### Histological analysis

For histological analysis, the tibias with implants were collected and fixed in 4% paraformaldehyde, and decalcification was performed for two weeks with 10% EDTA. The implants were then removed, and the proximal tibias (the area above the proximal end of the implant) were processed for paraffin embedding. The paraffin-embedded bone sections were subjected to TRAP staining, and osteoclastic parameters such as N.Oc/BS and Oc.S/BS were measured as described previously^52^. The images were collected with a microscope (Nikon ECLIPSE TE2000-S, Japan).

### Serum analysis of CTX-I and PINP

Eight weeks after implant insertion, blood was collected from the rats by heart puncture, and serum was separated by centrifugation (4000 rpm and 4 °C for 20 min). The serum CTX-I levels were analysed using a RatLaps EIA kit (IDS Nordic, Herlev, Denmark), and the serum PINP levels were measured using a Rat PINP ELISA kit (CUSABIO, Wuhan, China).

### Statistical analysis

All quantitative data are presented as the means ± SDs from three independent experiments. One-way ANOVA and Student-Newman-Keuls tests were used to determine significant differences in multiple comparisons, and p values less than 0.05 and 0.01 were considered significant and highly significant, respectively. All statistical analyses were performed with SPSS 17.0 software (SPSS, Chicago, IL, USA).

## Electronic supplementary material

Supplementary Information
