# Supplementary material for: Strontium-loaded titania nanotube arrays repress osteoclast differentiation through multiple signalling pathways: In vitro and in vivo studies
Source: Sci Rep. 2017 May 24;7:2328. doi: 10.1038/s41598-017-02491-9 (PMC5443803; doi:10.1038/s41598-017-02491-9)
Supplement: Supplementary file 1 — Supplementary Information [file 41598_2017_2491_MOESM1_ESM.pdf]

Supplementary Information

Strontium-loaded titania nanotube arrays repress osteoclast differentiation through multiple signalling pathways: *In vitro* and *in vivo* studies

Baoguo Mi[1], Wei Xiong[1], Na Xu[2, 3], Hanfeng Guan[1], Zhong Fang[1], Hui Liao[1], Yong Zhang[1], Biao Gao[2], Xiang Xiao[2], Jijiang Fu[2, 3*] and Feng Li[1*]

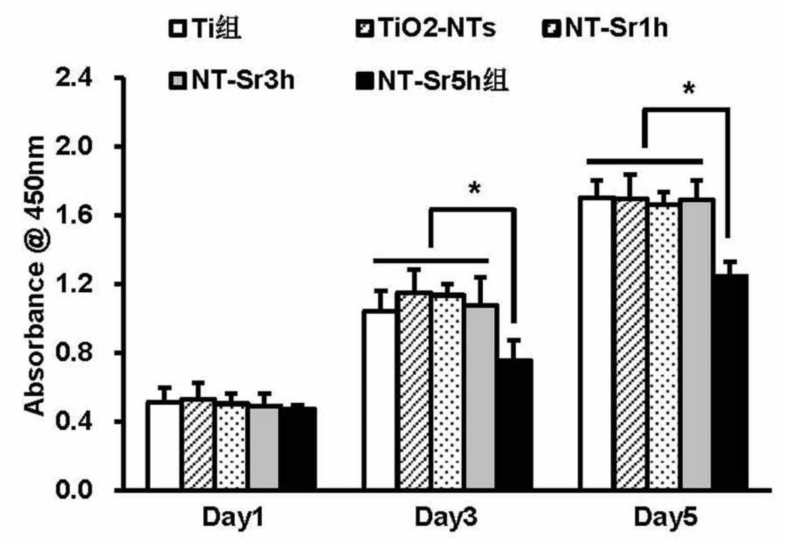

Supplementary Figure 1. Effects of the samples on cell proliferation.

RAW264.7 cells were cultured on samples (Ti, TiO_2-NTs, NT-Sr1h, NT-Sr3h, and NT-Sr5h) at a density of 2×10^4 cells per well for 1, 3, or 5 d, and the cell numbers were assessed using the Cell Counting Kit-8 assay. *$p < 0.05$, data are presented as the mean $\pm$ SDs ($n = 3$).

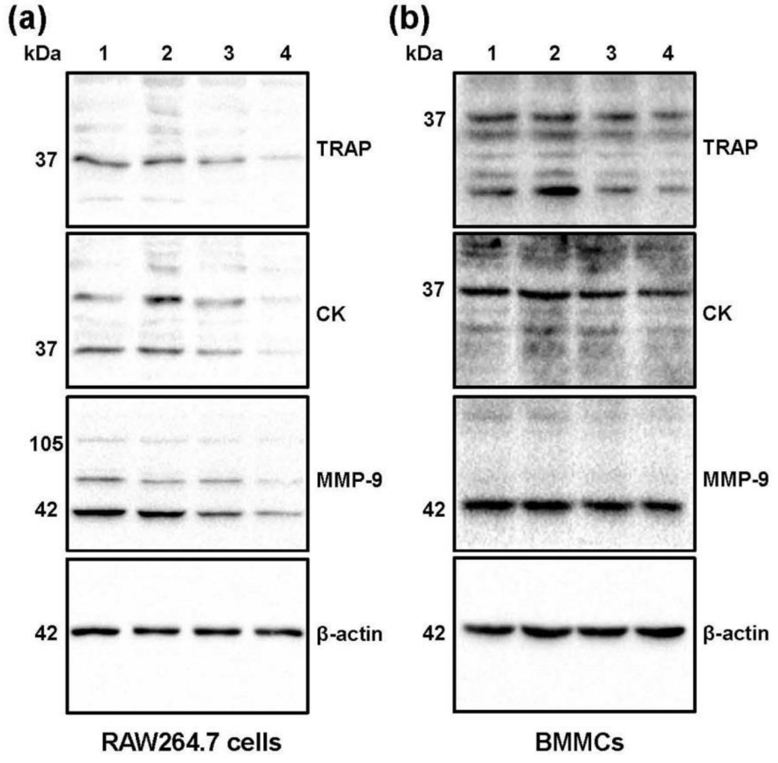

Supplementary Figure 2. NT-Sr repress osteoclast-specific genes.

RAW264.7 cells (a) and mouse BMMCs (b) were cultured on different samples and induced with 50 ng/mL RANKL and 30 ng/mL M-CSF (for BMMCs); then, the cells were collected for total protein. The protein expression of osteoclast markers (TRAP, CK, and MMP-9) was detected by immunoblotting. The antibody against β-actin was used as a loading control. The numbers 1, 2, 3 and 4 in the figure represent Ti, TiO_2-NTs, NT-Sr1h and NT-Sr3h, respectively.

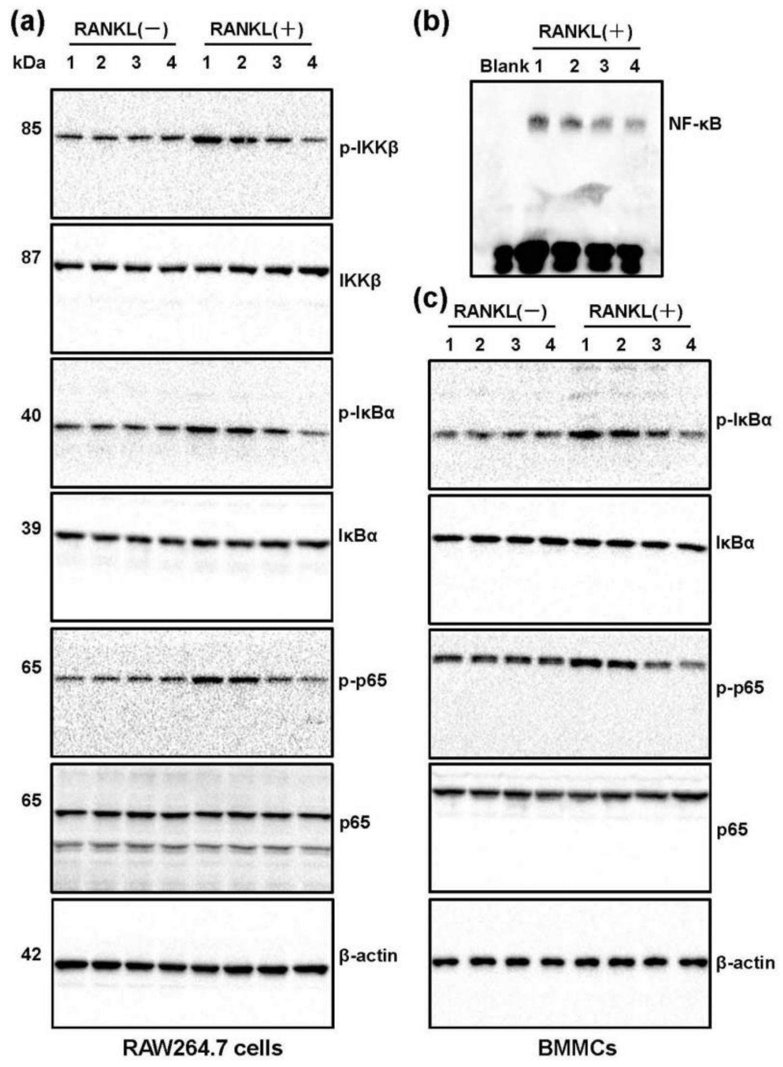

Supplementary Figure 3. NT-Sr inhibit RANKL-induced NF-κB activation.

RAW264.7 cells (a) and BMMCs (c) were cultured on different samples for 3 d; then, the medium was changed, and the cells were stimulated with or without RANKL (100 ng/mL) for 30 min, and total protein was extracted for western blot analysis. The expression of proteins in the NF-κB pathway and the levels of p-IKKβ, p-IκBα, and p-NF-κBp65 were determined. Antibodies to β-actin and total IKKβ, IκBα, and NF-κBp65 served as loading controls. (b) RAW264.7 cells were cultured on different samples for 3 d; then, the medium was changed, and the cells were stimulated with

RANKL (100 ng/mL) for 30 min. The nuclear extracts were prepared, and the DNA-binding activity of NF-κB was detected by electrophoretic mobility shift assay (EMSA). p-p65 and p65 represent p-NF-κBp65 and NF-κBp65; the numbers 1, 2, 3 and 4 in the figure represent Ti, TiO_2-NTs, NT-Sr1h and NT-Sr3h, respectively.

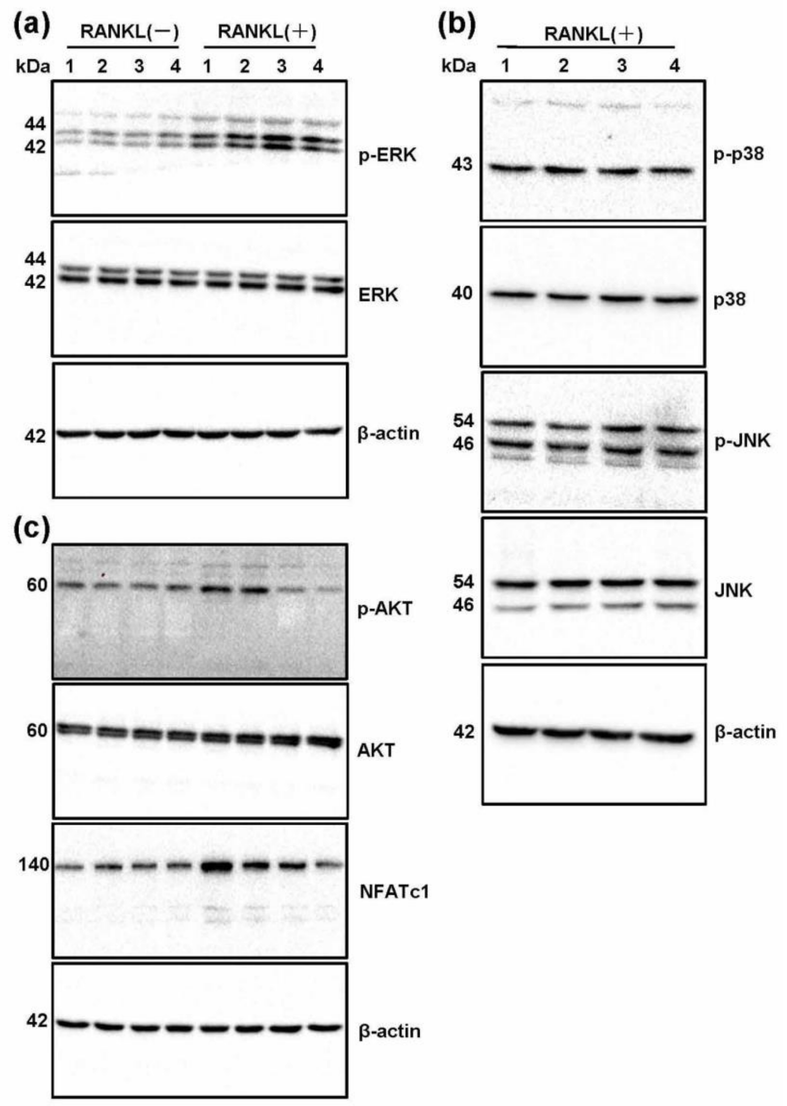

Supplementary Figure 4. The effect of NT-Sr on MAPKs and Akt/NFATc1 pathways.

RAW264.7 cells were cultured on different samples for 3 d, the medium was changed, and the cells were stimulated with or without RANKL (100 ng/mL) for 30 min. Then, total protein was extracted for western blot analysis. (a) RANKL-induced phosphorylation of ERK, (b) p38 and JNK were determined. (c) The expression of p-Akt and NFATc1 were determined. Antibodies to β-actin, total ERK, p38, JNK and Akt served as loading controls. The numbers 1, 2, 3 and 4 represent Ti, TiO_2-NTs, NT-Sr1h and NT-Sr3h, respectively.

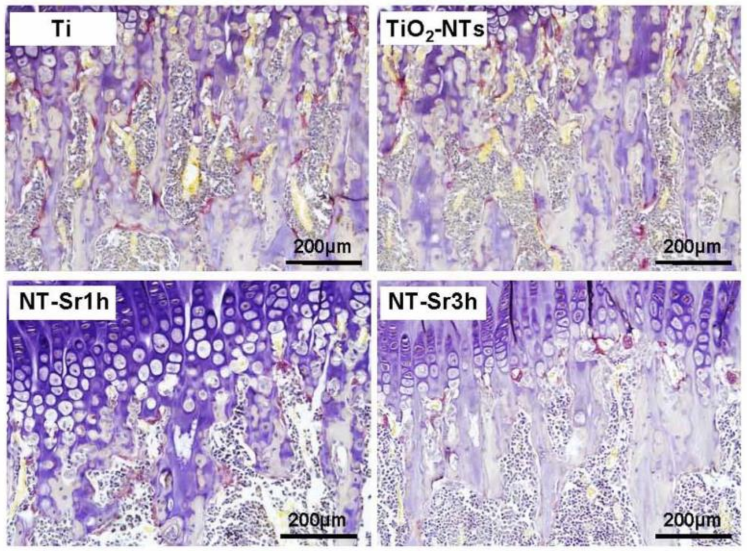

Supplementary Figure 5. NT-Sr decrease osteoclast formation in vivo.

OVX rats were sacrificed 8 weeks after implantation, sections of the metaphyseal regions of the proximal tibias (the area above the proximal end of the implant) were selected for TRAP staining. Red cells with three or more nuclei were identified as osteoclasts. The scale bar represents 200 μm.
